# Chemical Inhibition of Bacterial Protein Tyrosine Phosphatase Suppresses Capsule Production

**DOI:** 10.1371/journal.pone.0036312

**Published:** 2012-05-15

**Authors:** Alistair J. Standish, Angela A. Salim, Hua Zhang, Robert J. Capon, Renato Morona

**Affiliations:** 1 School of Molecular and Biomedical Science, University of Adelaide, Adelaide, South Australia, Australia; 2 Division of Chemistry and Structural Biology, Institute for Molecular Bioscience, The University of Queensland, St Lucia, Queensland, Australia; University of Edinburgh, United Kingdom

## Abstract

Capsule polysaccharide is a major virulence factor for a wide range of bacterial pathogens, including *Streptococcus pneumoniae*. The biosynthesis of Wzy-dependent capsules in both Gram-negative and –positive bacteria is regulated by a system involving a protein tyrosine phosphatase (PTP) and a protein tyrosine kinase. However, how the system functions is still controversial. In *Streptococcus pneumoniae*, a major human pathogen, the system is present in all but 2 of the 93 serotypes found to date. In order to study this regulation further, we performed a screen to find inhibitors of the phosphatase, CpsB. This led to the observation that a recently discovered marine sponge metabolite, fascioquinol E, inhibited CpsB phosphatase activity both *in vitro* and *in vivo* at concentrations that did not affect the growth of the bacteria. This inhibition resulted in decreased capsule synthesis in D39 and Type 1 *S. pneumoniae*. Furthermore, concentrations of Fascioquinol E that inhibited capsule also lead to increased attachment of pneumococci to a macrophage cell line, suggesting that this compound would inhibit the virulence of the pathogen. Interestingly, this compound also inhibited the phosphatase activity of the structurally unrelated Gram-negative PTP, Wzb, which belongs to separate family of protein tyrosine phosphatases. Furthermore, incubation with *Klebsiella pneumoniae*, which contains a homologous phosphatase, resulted in decreased capsule synthesis. Taken together, these data provide evidence that PTPs are critical for Wzy-dependent capsule production across a spectrum of bacteria, and as such represents a valuable new molecular target for the development of anti-virulence antibacterials.

## Introduction

Capsule polysaccharides (CPS) are fundamental virulence factors for a wide range of Gram-negative (e.g. *Klebsiella pneumoniae* and *Escherichia coli*) and Gram-positive (e.g. *Streptococcus pneumoniae* and *Staphylococcus aureus*) bacterial pathogens. Much work has been undertaken to investigate the regulation and mechanism of synthesis of this critical component of the cell, with our primary focus understanding the mechanism of regulation of the Wzy-dependent CPS of *Streptococcus pneumoniae* [1], [2], [3], [4], [5], [6], [7], [8], [9], [10], [11], [12], [13], [14], [15].

*Streptococcus pneumoniae*, commonly known as the pneumococcus, is a major human pathogen responsible for significant morbidity and mortality worldwide [16]. Both management and prevention of pneumococcal disease is becoming ever more difficult due to elevated rates of antibiotic resistance and increasing evidence of serotype switching and vaccine evasion to the current vaccine [17]. The additional lack of antibiotics in the development pipeline, makes the search for novel treatments of utmost importance [18].

The CPS is widely accepted as the major virulence factor of the pneumococcus, due to its ability to act as an anti-phagocytic factor [19], and is the target of currently used vaccines. To date, 93 different serotypes have been discovered [20], which makes coverage by the current vaccine severely limited, with protection provided against only 7 or 13 serotypes. Unencapsulated pneumococci are essentially avirulent and are unable to cause invasive pneumococcal disease, with mutations in CPS synthesis causing significant loss of virulence in animal models [6], [21], [22].

Biosynthesis of CPS in all but two pneumococcal serotypes occurs by a Wzy-dependent polymerization pathway, analogous to Group 1 CPS biosynthesis in *E. coli* and O-antigen assembly in Gram-negative bacteria [23]. The CPS biosynthesis loci of *S. pneumoniae* encode four genes (*cpsA-D* also known as *wzg, wzh, wzd & wze)* found at the 5′ end of the loci, which are involved in the regulation of CPS biosynthesis in the pneumococcus. Genes similar to these are found in the CPS loci of many other Gram-positive bacteria [24], [25], [26], [27]. While *cpsA* mutants produce significantly less CPS, the *cpsA* gene product is not essential for CPS production and is thought to function as a translational activator [28], [29]. *cpsC* encodes a PCP2b (polysaccharide co-polymerase) protein [30], and *cpsD* encodes an autophosphorylating protein-tyrosine kinase (PTK) [28]. CpsC- and CpsD-related proteins are found in both Gram-positive and Gram-negative bacteria [28], [31], [32]; in the latter they are fused into one protein (called a PCP2a protein) such as ExoP from *Sinorhizobium meliloti* [33] and Wzc from *E. coli* K-12 and K30 [11] (For recent reviews on PCPs see [32], [34], [35] ).

CpsB is metal-dependent protein tyrosine phosphatase (PTP) that is completely unrelated to any PTPs in eukaryotes, with homologues only found in other Gram-positive bacteria [7]. Interestingly, strains constructed with mutations in *cpsB* have produced different results, with some studies reporting lower levels of CPS [6], [7], [21], where others see an increase [8]. This has led to confusion about the role of the phosphorylation of CpsD and whether there is a positive or negative correlation of CpsD-P with CPS synthesis. Our hypothesis is that when CpsD is phosphorylated synthesis of CPS is enabled, whereas when de-phosphorylated by CpsB, the CPS is attached to the cell wall [6]. If correct this hypothesis would mean that mutants in both *cpsB* and *cpsD* would exhibit significantly lower levels of CPS, as either synthesis or attachment would be hindered. While there has been some discrepancy as to the affect defined mutations in *cpsB* have on CPS, all studies to date have shown that *cpsB* mutants are essentially avirulent in numerous animal models of infection [6], [8], [21]. Thus, CpsB represent a novel target for the development of anti-virulence drugs against *S. pneumoniae* and other Gram-positive pathogens.

Gram-negative bacteria such as *E. coli* [4], and *Klebsiella pneumoniae* [36] also possess PTPs that regulate CPS and exopolysaccharide biosynthesis. However, the representative PTP, Wzb, is not homologous to CpsB, but rather belongs to the family of low molecular weight protein tyrosine phosphatases [10], [37]. In *E. coli* K-12 and K30, deletion of the gene encoding Wzb results in no synthesis of colanic acid [1] (an exopolysaccharide produced by all *E. coli* isolates under stress conditions) and CPS [12], respectively. In other words, this PTP is thought to be essential for Gram-negative CPS synthesis.

The aim of this study was to identify chemical inhibitors of CpsB. To do so, we developed a screen in order to identify inhibitors of CpsB phosphatase activity. Using this assay, we discovered a compound (fascioquinol E; FQE) that could inhibit CpsB phosphatase activity both *in vitro* and *in vivo*. This inhibition consequently resulted in lower levels of CPS, and increased attachment of *S. pneumoniae* to a macrophage cell line. Furthermore, FQE also inhibited the *E. coli* PTP Wzb, and resulted in lower levels of CPS synthesis in *K. pneumoniae*. This suggests that the phosphatase activity of the PTPs CpsB and Wzb are essential for CPS production in *S. pneumoniae* D39 and Type 1 strains, and *K. pneumoniae* K1, respectively. FQE represents an attractive first step in the search for lead compounds that could be developed into “anti-virulence drugs”, which rather than targeting essential bacterial processes, target important virulence factors limiting the infectivity of the pathogen [38].

## Results

### Screening a Marine Extract Library for Inhibitors of CpsB Dephosphorylation of *p*-Nitrophenyl Phosphate

**Figure 1:**
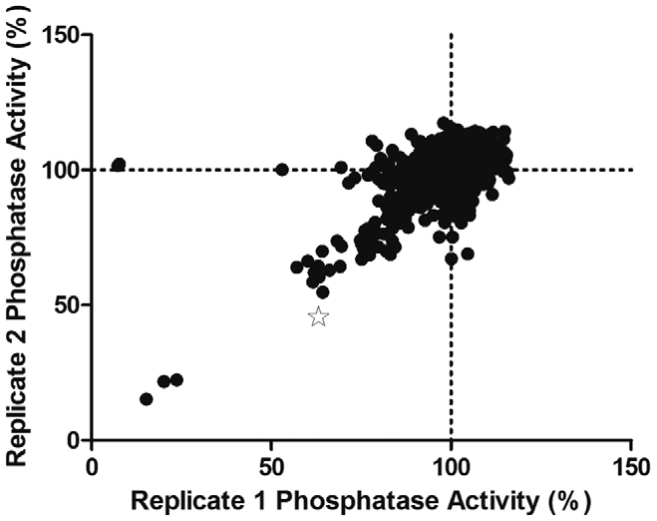
Screening of Marine Extract Library for inhibitors of CpsB activity. The ability of extracts to inhibit His_6_CpsB dephosphorylation of *p*NPP in 1 M Tris pH 8.0 with 1 mM MnCl_2_ was investigated in 96 well trays at 37°C. Shown is a plot of the two screening replicates reported as % phosphatase activity relative to the average of particular screening plate. The star represents the extract which produced the pure compound of interest.

We utilised the ability of CpsB to catalyse the dephosphorylation of *p*-nitrophenyl phosphate (*p*NPP) to develop an assay suitable for high throughput screening [7]. The reaction was linear, inhibited by broad phosphatase inhibitor sodium orthovanadate, and was dependent on MnCl_2_, while a mutated form of CpsB based on previous studies (CpsB^H5H7^) produced approximately 5% activity (data not shown) [39]. The assay produced a Z factor of >0.7, suggesting it was highly suitable for high throughput screening of inhibitors. Having optimised the CpsB assay, we used it to screen a Marine Extract Library comprising 2784 extracts derived from southern Australian and Antarctic marine invertebrates and algae. Each extract was screened in duplicate (see Figure 1) with high reproducibility revealing 17 extracts (0.6% hit rate) displaying greater than 30% inhibition of CpsB. In a proof-of-concept study we evaluated the CpsB inhibitory activity of a series of novel meroterpenes that had recently been isolated and reported from one of these priority extracts, generated from a deep-water southern Australian marine sponge *Fasciospongia* sp. (CMB-02028) [40].

### Fascioquinol E as a CpsB Inhibitor

**Figure 2:**
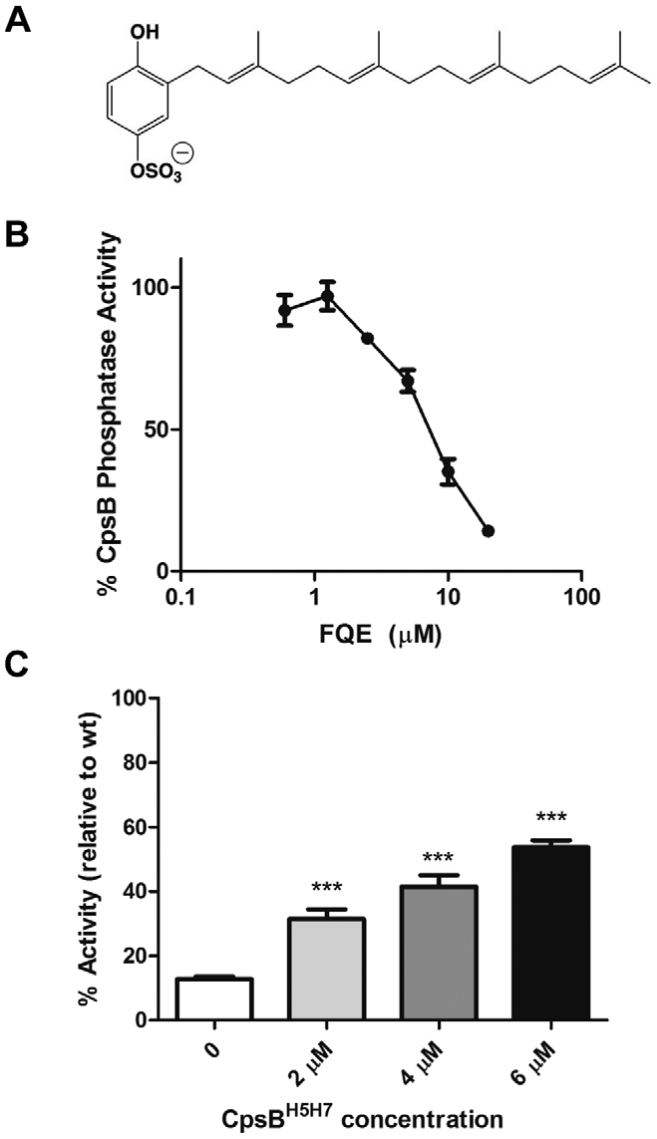
FQE inhibits CpsB dephosphorylation of *p*NPP. (A) Structure of FQE which (B) inhibited CpsB dephosphorlyation of *p*NPP with IC_50_ = 5.21 µM. (C) CpsB inhibition of pNPP dephosphorylation by FQE (10 µM) was investigated with increasing concentrations of CpsB^H5H7^. Data shown is from three independent experiments (*** - *P*<0.001 by Student’s *t*-test compared to no addition of CpsB^H5H7^).

In a prior 2011 investigation into the secondary metabolites produced by *Fasciospongia* sp. (CMB-02028), Zhang et al. [40] described six novel metabolites, fascioquinols A-F. On screening pure samples of fascioquinols A-F we established that fascioquinol E (FQE) was the dominant inhibitor of CpsB dephosphorylation of *p*NPP with an IC_50_ of 5.21 µM (Figure 2A & 2B).

In the 2011 report, FQE was noted as a modest Gram positive antibacterial (IC_50_ ≈ 3–5 µM) that was not cytotoxic against human gastric (AG) and colorectal (HT-29) adenocarcinoma, neuroblastoma (SH-Sy5Y) and human foreskin fibroblast (HFF-1) cell lines (IC_50_>30 µM) [40]. When we tested FQE antibiotic activity against *S. pneumoniae*, it inhibited the growth of D39 with an MIC (MIC = 3 µM) similar to that seen against other Gram-positive bacteria [40]. In order to determine if inhibition of CpsB activity was resulting in cell death, we also tested FQE against a D39 *cpsB* mutant. FQE also inhibited growth of this strain (MIC = 3 µM) with the same MIC, suggesting that inhibition of CpsB was not essential for its antibacterial effects. Controls with solvent alone showed no bactericidal activity.

In order to exclude that FQE was simply chelating manganese from the buffer (albeit unlikely as 1 mM Mn^2+^ was used), we performed the CpsB inhibitory assays with increasing concentrations of the inactive CpsB^H5H7^ protein while CpsB WT was incubated with FQE (10 µM). With increasing concentrations of CpsB^H5H7^, *p*NPP dephosphorylation by CpsB WT significantly increased, resulting in much less inhibition by FQE (Figure 2C). Thus, increasing concentrations of CpsB^H5H7^ competed away the inhibitory effects of FQE, suggesting that inhibition by FQE is competitive and that FQE inhibits the phosphatase by directly binding to CpsB.

### *In vivo* Effect of FQE on CpsD Tyrosine Autophosphorylation

**Figure 3:**
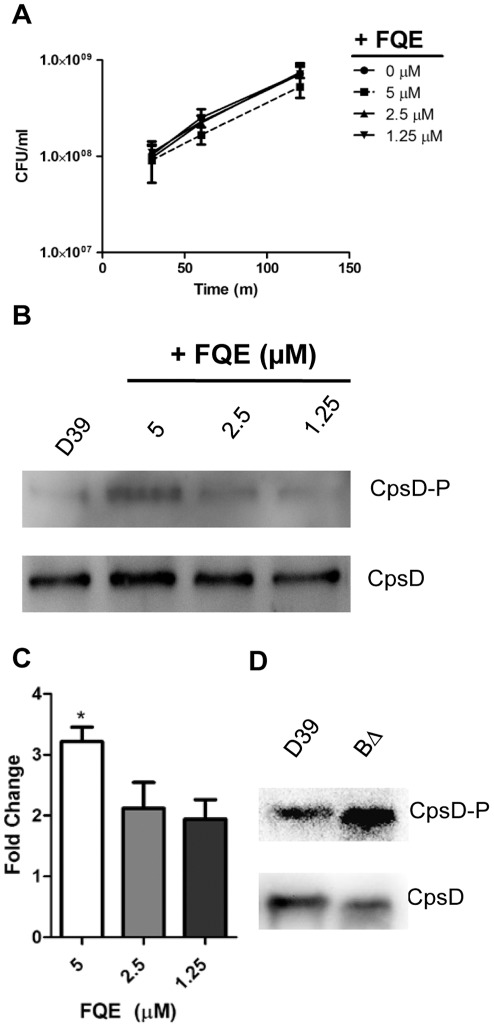
FQE increases CpsD-P in *S. pneumoniae* D39. *S. pneumoniae* D39 were grown to mid log phase in THY (OD_600_ ≈ 0.35) and FQE at indicated concentrations were added. (A) These concentrations (µM) had no statistically significant effect on CFU/ml after 30, 60 and 120 mins. (B) Whole cell lysates were prepared from these cells, which were separated by SDS-PAGE and analyzed by immunoblotting using anti-CpsD, or anti-phosphotyrosine (to detect CpsD-P). (C) Densitometric analysis of CpsD-P from three separate experiments. The effect with addition of 5 µM was significantly higher than compared with 1.25 µM FQE (* - *P*<0.05 by Student’s *t*-test). (D) For comparison, the effect of an in-frame *cpsB* deletion mutant on CpsD-P is shown.

While FQE inhibited the phosphatase activity of CpsB *in vitro*, we were interested to see if this would also inhibit activity *in vivo*. Thus, we grew D39 *S. pneumoniae* to mid log phase (OD_600_ ≈ 0.35) and addedFQE. A time course experiment showed that FQE had some effect on D39 CFU at 5 µM (although it did not reach statistical significance), but at 2.5 µM and below showed no growth inhibition (Figure 3A). As a read out of phosphatase activity of CpsB, we determined levels of CpsD-P in whole cell lysates made after one hour incubation with FQE, using Western immunoblot probing with anti-CpsD [28] and anti-phosphotyrosine. When grown in the presence of FQE, CpsD levels remained at similar levels to the untreated control (Figure 3B). However, the levels of CpsD-P increased by approximately 3 and 2 fold (Figure 3B & C) when incubated with 5 and 2.5 µM FQE, respectively. Thus, even when there was no impact on growth, FQE inhibited CpsB activity. This increase did not appear to be as much as seen in an otherwise isogenic D39*cpsB*Δ mutant (Figure 3D) [28], likely due to the residual activity of CpsB. However, this illustrated that FQE was able to inhibit CpsB phosphatase activity both *in vitro* and *in vivo*.

### *In vivo* Effect of FQE on Capsule Size

**Figure 4:**
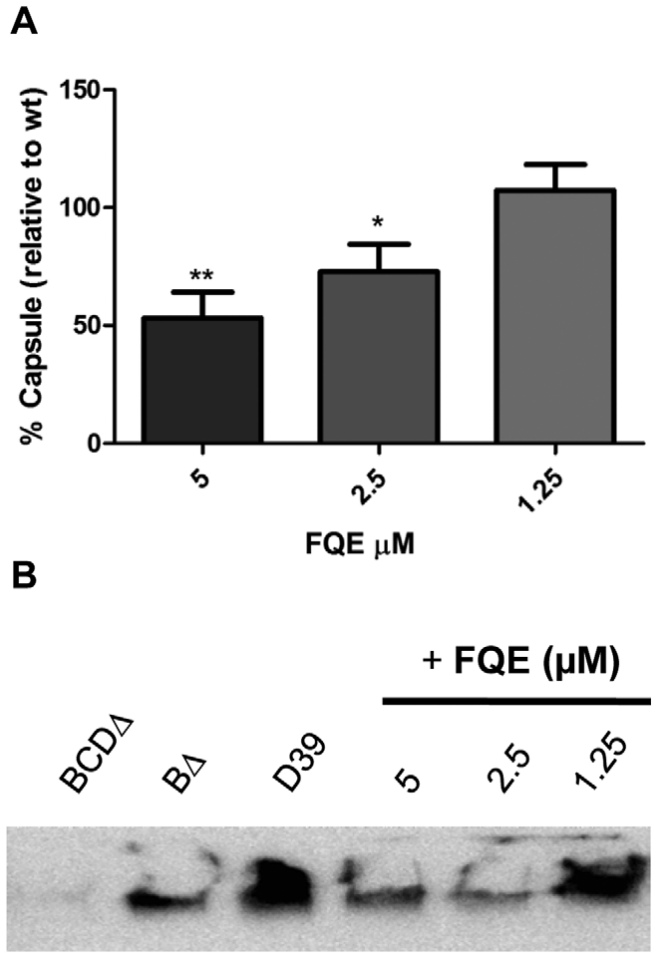
FQE decreases capsule synthesis in *S. pneumoniae* D39. Total CPS preparations were isolated from equal numbers of bacteria after incubation with FQE for 1 h. CPS levels were analysed by either (A) uronic acid assay or alternatively (B) by separating CPS on SDS-PAGE, transferring to Nylon and the probing with α-cps2 as described in the materials and methods. Data in (A) is from ≥3 independent experiments (5 µM vs 1.25 µM; * - *P*<0.05 by Student’s *t*-test).

With FQE affecting the tyrosine phosphorylation of CpsD, we wanted to see if this resulted in a subsequent reduction in CPS. The first method utilized was the colorimetric uronic acid assay [41], as glucuronic acid is a component of the Type 2 repeat unit [20]. This assay showed that CPS synthesis was reduced by approximately 47% with 5 µM and 28% with 2.5 µM FQE (Figure 4A). When incubated with 1.25 µM FQE, uronic acid levels did not decrease. Additionally, CPS preparations were separated on SDS-PAGE, transferred to nylon and probed with a polycolonal antibody against Type 2 CPS. This showed similar results to those seen with the uronic acid assay, with reduction of CPS levels at 5 and 2.5 µM, but no effect at 1.25 µM (Figure 4B). Control strains D39*cpsBCD*Δ and D39*cpsB*Δ showed reductions as previously reported [21].

We also tested FQE against a *S. pneumoniae* serotype 1 invasive clinical isolate. Serotype 1 possesses galacturonic acid in its CPS, allowing us to measure FQE mediated affect on CPS by the uronic acid assay again. Incubation with 5 and 2.5 µM FQE resulted in 38% ±9.6 and 30% ±18 reductions in uronic acid respectively (n = 4). Thus, this data suggested that FQE mediated inhibition of CpsB phosphatase activity resulted in lower levels of CPS synthesis in *S. pneumoniae*.

### FQE Treatment Increases Attachment of Pneumococci to Macrophages

**Figure 5:**
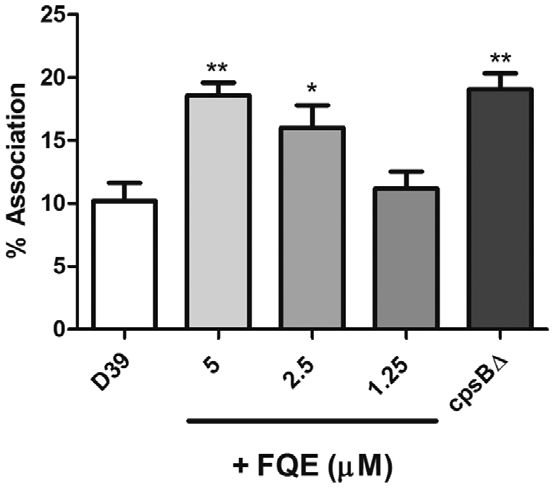
FQE increases attachment of D39 to macrophages. D39 was incubated with FQE and then assessed for its ability to associate with RAW 264.7 cells as described in Materials and Methods. Data is presented as % association relative to inoculum. D39*cpsB*? was used for comparison purposes. Results are from three independent experiments (** - P<0.01; * - P<0.05 compared to D39 by Student’s *t*-test).

The CPS of *S. pneumoniae* is primarily thought to be critical through its ability to act as an anti-phagocytic factor [19]. Additionally, unencapsulated pneumococci show increased adherence to a variety of cell types [6]. Thus, we sought to investigate whether FQE could affect the ability of pneumococci to associate with the murine macrophage cell line, RAW 264.7. D39 was incubated with 5, 2.5 and 1.25 µM FQE for 1 h as described above, and association with the macrophage cell line was determined as outlined in the methods. Concentrations of FQE (5 and 2.5 µM) that inhibited CPS production (Figure 4A) also significantly increased the association of D39 with RAW 264.7 cells (5 µM – *P*<0.01; 2.5 µM – *P*<0.05) (Figure 5). This was comparable with the increased association seen with an otherwise isogenic D39*cpsB*Δ mutant.

### FQE also Inhibits Wzb and Gram-negative Capsule Synthesis

**Figure 6:**
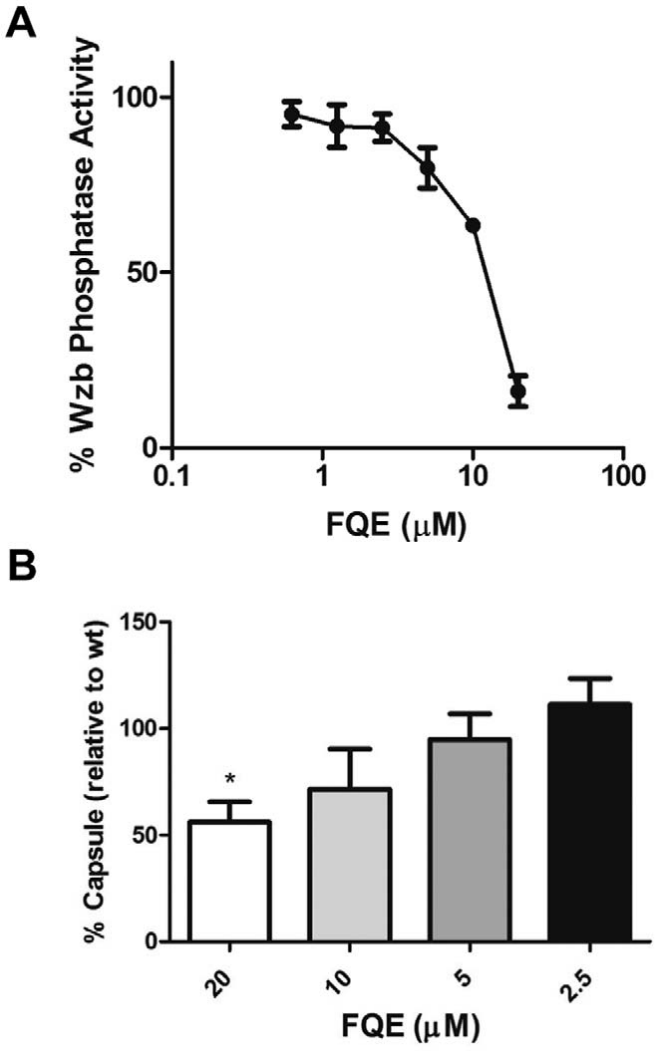
FQE also inhibits *E. coli* Wzb and CPS synthesis in *K. pneumoniae O1*. (A) His_6_Wzb dephosphorylation of *p*NPP in presence of FQE in 1 M Tris pH 7.0 at 37°C. (B) Total CPS preparations from *K. pneumoniae* incubated with FQE were analysed by uronic acid assay. Data is from four independent experiments (20 µM vs 5 µM; * - *P*<0.05 by Student’s *t*-test:).

As previous data had shown that CpsB was able to act on the Gram-negative PTK Wzc [10], this suggested that CpsB and the PTP from *E. coli*, Wzb, showed significant similarity in their active sites [10]. Thus, we investigated if FQE could also inhibit Wzb’s ability to catalyze dephosphorylation of *p*NPP. Interestingly, FQE inhibited the activity of purified Wzb with a similar IC_50_ as CpsB (Figure 6A), suggesting that FQE may also be able to inhibit CPS production in Gram-negative bacterial pathogens.

*Klebsiella pneumoniae* is a Gram-negative pathogen which causes primarily nosocomial infections. The pathogen possesses highly similar homologs to Wzb and Wzc from *E. coli* [36]. Additionally, the CPS has been shown to be critical for its ability to cause invasive disease [42]. Thus, we investigated whether FQE could inhibit CPS production in *K. pneumoniae* as well as in the pneumococcus. *K. pneumoniae* K1 [43] was grown to mid-log phase (OD_600_ ≈ 0.4) and then incubated with FQE for 1 h. The uronic acid colorimetric assay was used to quantify CPS as K1 serotype CPS possesses glucuronic acid as a component of its CPS [44]. As FQE does not inhibit the growth of Gram-negative bacteria, we were able to utilize it at higher concentrations [40]. FQE was also able to inhibit CPS synthesis in *K. pneumoniae*, although approximately 5 fold more inhibitor was required (20 µM) (Figure 6B). The latter was not unexpected, as the presence of the outer membrane of Gram-negative bacteria confers decreased permeability to very small molecules. Thus, this result indicated that inhibition of Wzb in Gram-negative bacteria also results in reduced CPS production.

## Discussion

Capsular polysaccharide is a crucial virulence determinant for a wide range of bacterial pathogens, both Gram-positive and -negative. Interestingly, its regulation is similar across both genera, with a PTP and a PTK controlling synthesis of one major class of CPS. We are particularly interested in the regulation of its synthesis in the major human pathogen, *Streptococcus pneumoniae*.

The study of this system in *S. pneumoniae* and other bacterial pathogens has to date been confined to the use of otherwise isogenic strains containing mutations in the various genes comprising the regulatory system. However, this is not ideal, as the regulatory locus is comprised of an operon, with mutations potentially resulting in subtle unanticipated effects. Thus, in order to study the system using alternate methods, we set out to discover inhibitors of the *S. pneumoniae* PTP, CpsB. Utilising the ability of CpsB to dephosphorylate *p*NPP, we performed a screen of a marine extract library culminating in the discovery of a novel meroterpene sulphate, FQE, which inhibited CpsB phosphatase activity with an IC_50_ of 5.21 µM. FQE had previously been shown to have antibacterial effects against Gram-positive but not Gram-negative bacteria [40], but this activity appeared unrelated to CpsB as it was able to inhibit the growth of a *cpsB* mutant.

Using FQE at concentrations where bacterial growth was not significantly inhibited, we showed that incubation of *S. pneumoniae* D39 with FQE resulted in increased levels of CpsD-P, but not CpsD levels itself. This suggested that FQE penetrates the cell and inhibits CpsB phosphatase activity, at concentrations that do not affect growth. Furthermore, we saw a significant decrease in CPS production when both D39 and a Type 1 strain were incubated with FQE. This suggested that full activity of the PTP CpsB is essential for the ability of the pathogen to produce a fully encapsulated cell. Additionally, at levels which resulted in decreased levels of CPS synthesis, we saw increased attachment of D39 to the mouse macrophage cell line, RAW 264.7, similar to the levels seen with an otherwise isogenic mutant in *cpsB*. Attachment to macrophages is crucial for the ability of the host to clear pneumococcal infection [45], and the CPS is a crucial factor in this anti-phagocytic ability [19]. Thus, this suggested that the PTP CpsB is crucial for encapsulation, and the subsequent full virulence of the pathogen.

Previous studies using deletion knockout mutants have provided conflicting results as to whether CpsB activity is essential for CPS synthesis. We have seen in numerous strain backgrounds that CpsB is required for the full expression of CPS through the use of *cpsB* mutants [6], [7], [21], while Bender et al. (2003) saw a slight increase in CPS in D39 [8]. Additionally, we showed that deletion of the phosphorylated tyrosine residues at the C-terminus of CpsD also resulted in an unencapsulated bacterium [46]. The results presented here support the conclusion that CpsB function is critical for complete synthesis of pneumococcal CPS. Interestingly, a recent study has suggested a novel role for CpsC and CpsD in the synthesis of CPS at the division septum [47]. This study did not investigate whether CpsB also plays a similar role, although this seems unlikely as the C-terminal cluster of tyrosines in CpsD was not required. Thus, this suggests that there may be multiple methods of regulation controlling CPS production in the pneumococcus (septal and non-septal).

FQE also inhibited activity of the PTP from *E. coli*, Wzb, at similar levels to that seen for CpsB. While CpsB and Wzb show no structural similarity, a recent study compared the PTPs and found that they shared common chemical features, explaining why CpsB can dephosphorylate Wzc, and, in our case, FQE can inhibit both PTPs [10]. FQE is not a simple promiscuous phosphatase inhibitor as it is unable to inhibit another phosphatase (Shrimp Alkaline Phosphatase) at concentrations up to 200 µM (data not shown). The inhibition of Wzb prompted us to investigate whether FQE could also inhibit Gram-negative CPS synthesis in *K. pneumoniae*, an important nosocomial human pathogen that has a PTP homologous to Wzb [36]. Incubation of *K. pneumoniae* K1 with FQE resulted in lower levels of CPS synthesis, suggesting that activity of the PTP in Gram-negative bacteria is also important for complete CPS synthesis. Furthermore, as FQE had no effect on the growth of Gram-negative bacteria, this result gives further support to a direct inhibition of PTP. Other studies have shown that in *E. coli* expression of Wzb is critical for CPS expression [12], and that the extent of phosphorylation of the PTK influences the amount of CPS produced [11]. Thus, this study provides further credence to these results and reinforces the importance of Wzb in CPS biosynthesis.

The small molecule inhibition (FQE) of PTP activity in both a Gram-positive and -negative pathogen leading to lower levels of CPS provides strong evidence that these PTPs are suitable targets for the development of an anti-virulence drug. Such a class of anti-virulence therapeutics would differ from conventional antibiotics in that they would not inhibit the growth of the bacteria but would suppress virulence and down-regulate the intensity and impact of any infection. While it is generally accepted that anti-virulence antibacterials would invoke less selective pressure on bacteria, it is important to consider that the critical nature of CPS *in vivo*, such as through resistance to opsonophagocytosis as well as in competition with other microbes [48], may result in the selection of pneumococci resistant to drugs such as FQE. However, with the ever increasing need for novel anti-microbials, we have shown that the conserved capsule regulatory system appears to be a promising target. We are currently working on optimizing the FQE CpsB inhibitory pharmacophore, and investigating the additional priority hits detected in our screening program, with a view to discovering and developing more potent inhibitors of Wzb and CpsB activity.

## Materials and Methods

### Growth Media and Growth Conditions

*S. pneumoniae* D39 [49] and type 1 (WCH 4496) [50] were grown in Todd-Hewitt broth with 1% Bacto yeast extract (THY) and C+Y [51] respectively, or on blood agar. Agar plates were grown at 37°C in 5% CO_2_. Broth cultures were grown at 37°C without agitation. *Escherichia coli* strains and *K. pneumoniae* Kpn1 [43] were grown in Luria-Bertani broth (10 g/L Tryptone, 5 g/L yeast extract, 5 g/L NaCl) broth or agar, with transformation carried out using CaCl_2_-treated cells. D39*cpsB*Δ and D39*cpsBCD*Δ were previously described [6].

### Expression and Purification of His_6_CpsB, His_6_BH5H7 & His_6_Wzb

CpsB from TIGR4 cloned under control of a pBAD promoter (pWQ553) was transformed into *E. coli* Lemo21(DE3) [52]. 6× HisCpsB expression was induced by induction for 3 hours 0.1% (w/v) arabinose. The soluble recombinant protein was purified using an AKTA prime plus (GE Life Sciences) with a HiTrap column as described by the manufacturer. The protein was concentrated using Vivaspin 6 (GE Healthcare). The protein was stable in 50% (v/v) glycerol. His_6_CpsB^H5H7^ was purified using the same method. His_6_Wzb was expressed and purified as described previously [12].

### Construction of CpsB^H5H7^

H5 and H7 of CpsB from pWQ553 were mutated to alanine using QuikChange® Site Directed mutagenesis kit (Stratagene) according to the manufacturer’s instructions. Oligonucleotides used were AS50 (ATGATAGACATCGCATCGGCAATCGTTTTTGATG) and AS51 (CATCAAAAACGATTGCCGATGCGATGTCTATCAT).

### *p*-Nitrophenyl Phosphate Dephosphorylation

His_6_CpsB catalysis of *p*NPP (1.5 mM) (Sigma) dephosphorylation was carried out in 100 µl of 1 M Tris pH 8.0 with 1 mM MnCl_2_ in 96 well flat bottom tray (Corning) [7]. Reactions were incubated at 37°C with A_410_ recorded every minute on PowerWave XS (Biotek). After 10 min, change in absorbance was calculated. The Z’ was calculated using a previously published equation [53]. Catalysis using His_6_Wzb was carried out using the same method, however buffer was 1 M Tris, pH 7.0 and 100 nM His_6_Wzb was used.

### Natural Product Extract Partitioning and Fractionation

Marine algae and invertebrate samples were collected from southern Australian and Antarctic waters between 1984–2002. No specific permits were required for the described field studies. The freshly collected samples were frozen (−4°C) for shipping to the laboratory, where they were thawed, catalogued, diced, and steeped in aqueous ethanol for prolonged storage in −20°C. A portion of the ethanol extracts were dried by rotary evaporation (<40°C) and partitioned between *n*-butanol and water. The *n*-butanol extracts were dried and made up to a standard concentration, and were screened in the CpsB assay by measuring the inhibition of *p*NPP dephosphorylation. One active extract, generated from the *Fasciospongia* sp. sponge has been studied in detail [40]. Screening of the pure compounds isolated from *Fasciospongia* sp. led to the identification of fascioquinol E (FQE) as the most active compound.

### Western Immunoblotting

Whole cell lysates from equal numbers of cells or CPS preparations were separated on 12% SDS-PAGE and transferred to Immobilon-P (Millipore) (anti-Phosphotyrosine, Santa Cruz Biotechnology catalog no. sc-7020), Nitrobind (GE Water and Process Technologies) (anti-CpsD [28]) or Hybond-N (Amersham)(anti-Cps2 (Statens Serums Institut)). Membranes were probed with primary antibody overnight and after washes incubated as appropriate either with horseradish peroxidase-conjugated goat anti-rabbit or goat anti-mouse secondary antibodies (Biomediq DPC) for 2 h. The membrane was then incubated with chemiluminescence blotting substrate (Sigma) for 5 min. Chemiluminescence was detected by Kodak Image Station 4000 MM Pro.

### Uronic Acid Assay

The quantitative uronic acid assay [41] was undertaken for *S. pneumoniae* D39 and type 1 as described previously [6] with CPS preparations from cultures grown in THY and C+Y respectively. All samples were equilabrated such that CPS was being determined for equal number of cells from each sample. For *K. pneumoniae*, the uronic acid assay and CPS preparations were undertaken according to the method previously described [54]. Briefly, samples (500 µL) of bacterial cultures were removed and mixed with 100 µL of 1% Zwittergent 3–14 detergent (Calbiochem, Meudon, France) in 100 mM citric acid (pH 2.0). This mixture was incubated at 50°C for 20 min. After it was centrifuged for 5 min at 14,000 rpm, 300 µL of the supernatant was transferred to a new tube and absolute ethanol was added to a final concentration of 80%. The mixture was placed at 4°C for 20 min. After centrifugation (14,000 rpm), the supernatant was decanted and the pellet was dissolved in 200 µL of distilled water.

### Cell Association Assay

RAW 264.7 (murine macrophage-like) cells (ATCC; Catalog number TIB-71) were grown to confluence in a 24 well tissue culture plate (Nalge Nunc International) (approximately 18 h) at 37°C, 5% CO_2_. Bacteria grown to mid-log phase at 37°C with aeration were washed once with PBS and resuspended in RPMI or DMEM (without supplements) as appropriate. Tissue culture cells were washed once with fresh media and 500 µL of the appropriate supplemented media added. 100 µL of undiluted bacterial suspension was added to each well and a sample retained to determine the inoculation dose. Plates were centrifuged at 500×g for 5 min to increase interaction of bacteria and cells and incubated for 30 min at 37°C, 5% CO_2_. Wells were washed three times with fresh media and 100 µL 0.1% (v/v) Triton X-100 added for 10 min at RT to lyse the eukaryote cell membranes. 400 µL PBS was added to the wells and the number of viable bacteria determined by culturing on selective media. Results were expressed as mean and standard variation and statistical difference assessed by unpaired two-tailed student t-test.

### Antimicrobial Growth Assay

*S. pneumoniae* D39 was inoculated into broth and then incubated with FQE at a range of concentrations in THY at 37°C in a 96 well tray sealed with Breath easy membrane (Sigma) in Powerwave XS. A_600_ readings were taken every 20 min for 16 h.
